# Pre-surgical socket analysis for immediate implant placement

**DOI:** 10.4317/medoral.26269

**Published:** 2024-02-18

**Authors:** Francisco Javier Parra-Moreno, Verónica Schiavo-Di Flaviano, Sonia Egido-Moreno, Constanza Saka-Herranz, Albert Estrugo-Devesa, José López-López

**Affiliations:** 1 Master of Medicine, Surgery and Oral Implantology of Faculty of Medicine and Health Sciences (Dentistry) and University of Barcelona Dental Hospital (HOUB), University of Barcelona, L'Hospitalet de Llobregat, Barcelona, Spain; 2 Department of Odontoestomatology, Faculty of Medicine and Health Sciences (Dentistry), University of Barcelona, L'Hospitalet de Llobregat, Barcelona, Spain; 3 Oral Health and Masticatory System Group, IDIBELL (Bellvitge Biomedical Research Institute), Barcelona, Spain; 4 Facultative Director and Head of Service of the Medical-surgical area of the Dental Hospital of the University of Barcelona, Spain

## Abstract

**Background:**

Traditional protocols for implant surgery suggest a healing period of 2-3 months from dental extraction to implant placement. Based on all the volumetric modifications produced by that approach, there are authors who advocate for immediate implantology. The aim of the present study was to determine the prevalence of different sockets, and the dimensions of the bone around the upper anterior incisors and canines, to determine the predictability of immediate implants in our population.

**Material and Methods:**

This is an observational, cross-sectional study based on cone-beam computed tomography images of the anterior maxila of patients attending the Odontological Hospital of the University of Barcelona (OHUB) and requesting for implant treatment. Different measurements were performed on every analyzed tooth, and also they were categorized by using the main dental sockets classifications.

**Results:**

Bone attachment levels and cortical thickness are lower in women compared to men in all three types of teeth (the difference in the bone attachment levels ranges from 4.68%-8.63% and in the bone thickness goes from 0.02-0.58mm). Bone attachment level gradually reduces with age. The reductions observed in all the measurements are higher in the case of canines, compared with the other teeth. The differences from patients <45 years old and patients between 55-64 years old are 13.58±14.55mm in the case of central incisors, 10.04±5.52 in the case of lateral incisors and 22.39±13.65mm in the case of canines.

**Conclusions:**

According to our results, the canines are the teeth with the greatest complexity when it comes to immediate implantology treatments. Furthermore, that kind of treatment is more complex as age increases, since we observed a gradual percentage of unfavourable sockets in older patients.

** Key words:**Bone attachment, cortical bone height, cortical thickness, bone resorption, type of socket, immediate implants.

## Introduction

Usually, dental extractions lead initially to a partial loss of the teething, which over time, can result in edentulism. This can have an impact on oral and general health (1). Endosseous implants have become a valuable alternative to dental prostheses supported by remaining teeth or adjacent oral soft tissues (2). Nevertheless, complications as failures and diseases such peri-implantitis are not uncommon and its success rate ranges from 95% to 98% (3).

When a dental extraction is performed, there are a number of steps which are necessary for the healing process. The first step is the filling of the socket with a reinforced clot covered by a fibrin mesh. On the third day, this clot transforms into granulation tissue that matures by the 7th day, becoming a provisional matrix which replaces the primary clot. By 14th day, the socket is filled by a dense trabeculae, progressing to a reticular bone tissue which fills approximately one third of the socket. By day 90, this reticular bone tissue has transformed into dense lamellar bone, while soft tissue closure occurs between the 4th and 6th week (4,5). In most cases, during the healing process, the alveolar bone presents a 50% decrease in thickness in the first year. Horizontal and vertical bone loss after 6 months are 29-63% and 11-22% respectively (6,7). One study conducted by Marconcini *et al*. (8), emphasized the paper of myofibroblast on socket healing after extraction. They have a major role on the overall remodeling pattern of the alveolar bone. As they showed, myofibroblast disappear when overlying epithelial closure is achieved, which takes place after 15 days in the oral mucosa.

Traditional protocols suggest a healing period of 2-3 months from dental extraction to implant insertion. However, the negative effect of dental extraction on the volumetric changes of hard and soft tissues. Based on all these changes in the post-extraction socket and trying to prevent that bone resorption, there are some authors who advocate for immediate implants. Other advantages of this technique in aesthetic areas are the reduction in the number of surgeries and the total treatment time (9,10).

On the other hand, It is well known that to ensure the success of an immediate implant, intact bone walls and the absence of an active infection are required. Primary stability must be achieved by anchoring the implant to the palatal wall and inserting it 4-5mm in the apical bone (11,12).

The classifications of the types of post-extraction sockets are intended to help the clinician in the diagnosis, carry out a correct therapeutic orientation, and anticipate the need to use biomaterials. Moreover, they can help us know whether the treatment by immediate implantology is predicTable (12,13).

The objective of this cross-sectional observational study was to determine the prevalence of the different types of sockets according to multiple classifications and analyzing the bone dimensions around the upper anterior incisors and canines. In the same way, the different positions of the dental roots were taken into account. Variations depending on age and sex were taken into account. Likewise, the clinical implications of the results obtained were discussed.

## Material and Methods

- Study sample

The sample was obtained from patients for whom a CBCT (Cone Beam Computed Tomography) of the anterior maxilla was required for their treatment and who had at least a remaining maxillary central incisor, lateral incisor or canine.

CBCT images were obtaines from Planmeca® ProMax3DMid Proface CBCT engine and were displayed and analyzed using Planmeca Romexis® software. The technical specifications are as follows: voxel size of 200𝛍m, 90kV and 10mA.

The study protocol was approved by the standing ethics committee of the Faculty of Dentistry, University of Barcelona (Comité d’Ètica de l’Hospital Odontològic ude.bu@cigolotnodolatipsoh.ciec) (Number 42/2022) and was conducted as a cross-sectional observational study in accordance with the Taipei Declaration (version 2016) and the STROBE statement guide (14).

- Study population

The study population consisted in patients who attended Hospital Odontològic Universitat de Barcelona (HOUB) and underwent a CBCT that included the upper anterior area.

Inclusion / exclusion criteria: Within inclusion criteria we included subjects over 18 years old, patients with the presence of 1 or more teeth from the upper right canine to the upper left canine at the time of performing the CBCT, and they must include the superior anterior portion. Moreover, no surgeries on the area prior to the CBCT (hard and/or soft tissue) should have been performed. The exclusion criteria were subjects under 18 years old, absence of upper anterior teeth, CBCT´s which do not include the superior anterior portion, patients with a history of treatments on hard and/or soft tissues surrounding teeth in the area of study, teeth with endodontic treatments, with prosthetic crowns and veneers, those subjected to orthodontic forces, or with fractured or resorbed roots, in addition to patients presenting upper anterior dental crowding.

- Sample size

Taking as reference the article by Gluckman *et al*. (15), it is concluded that the sample size should be 150 CBCTs. This is a convenience sampling, where the CBCTs available in the database of the OHUB were included until the necessary sample size is reached.

Moreover, considering the population that comes to the HOUB to undergo an implant treatment of the area of study, we calculated that the adequate sample to achieve a confidence level greater than 95 %, should be 149 patients.

- Procedures

The following main variables were analyzed:

Sex: categorical-dichotomous

Age: numerical-continuous

The following measurements were made:

Rooth length: numerical-continuous

Buccal and palatal bone height: numerical-continuous

Apical bone height: numerical-continuous

Buccal, palatal and post-apical cortical thickness: numerical-continuous

Those measurements allowed the classification of the sockets into different indices/values (Table 1).

**Figure 1:**
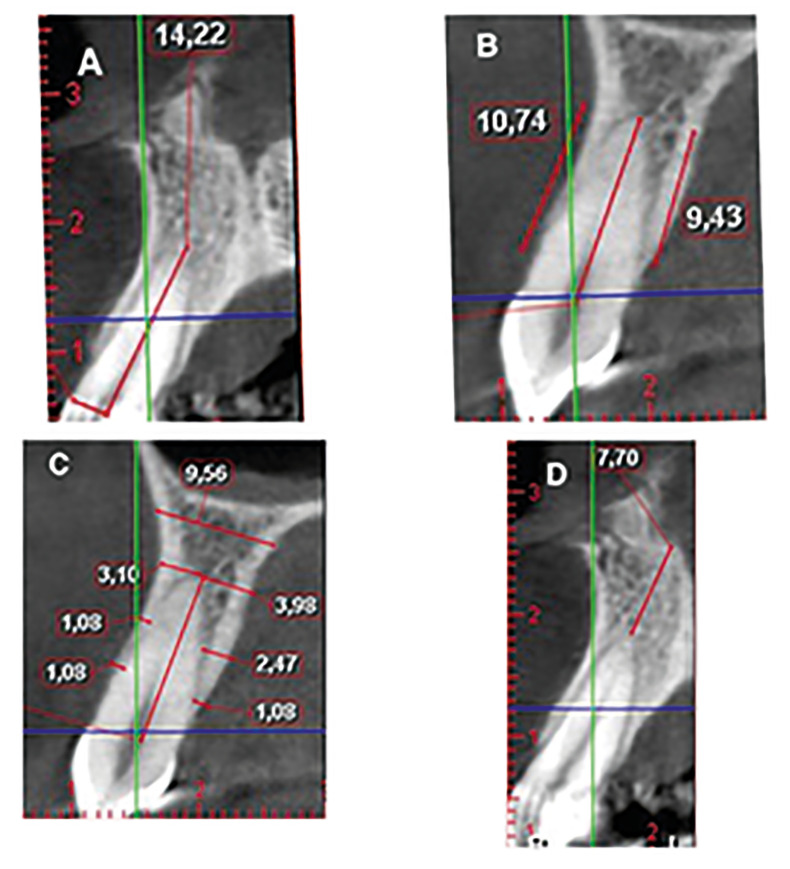
Measurements taken on every CBCT image. (A) Method of measuring the root length. Measurement of the distance from the CEJ projected on the midpoint of the root on a sagittal vision, to the point of the root apex. (B) Method of measuring the buccal and palatal bone height.A line is drawn from the most coronal point (bone peak) of the buccal and palatal cortical, to the point of the root apex on both buccal and palatal sides. (C) Method of measuring the thickness of the buccal and palatal corticals. Three lines were drawn perpendicular to the sagittal axis of the root, both buccal and palatal: line A: 1mm apical to the alveolar crest, line C: at the apical point, and line B: in the middle distance between points A and C. At 4mm apical from the root apex, the post-apical line is drawn from buccal to palatal sides. (D) Method of measuring the apical bone height. A line is drawn from the apex of the tooth to the floor of the nostril.

Root length: This measurement consisted in the distance from the CEJ (cemento-enamel junction) projected over the midpoint of the root to the root apex, seen in the sagittal projection of the CBCT (Fig. 1).

Measurement of buccal and palatal bone height: The bone height was determined by means of the image obtained from the CBCT. This measurement was taken from the most coronal point (bone peak) of the buccal and palatal cortical, to root apex. Both lines were parallel to the root length line. Distances equal to or less than 1.07mm between the CEJ and the alveolar crest were considered physiological. This value was provided by the literature (16) and made it possible to calculate the percentage of remaining bone attachment (Fig. 1).

Measurement of buccal and palatal bone thickness: This measurement was made on 4 points in the sagittal projection of the CBCT. Firstly, it was drawn on three points in both the buccal and palatal cortical:

Line A: 1mm apical to the alveolar crest. Line C: at the apical point. Line B: between points A and C. Finally, 4mm apical from the root apex, the post-apical line was drawn from buccal to palatal surfaces. All these lines are perpendicular to the root surface (Fig. 1).

**Table 1:**
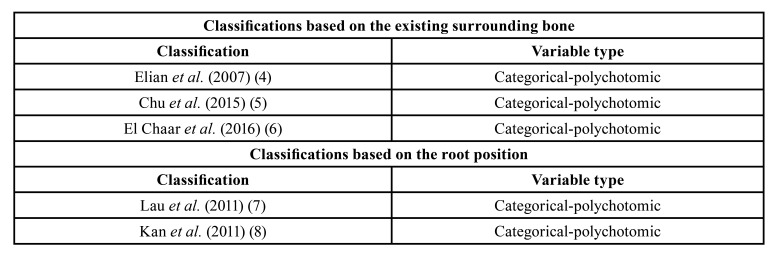
Different socket classifications used for the study.

Measurement of apical bone height: The height from the apex of the tooth to the floor of the nostril was determined by sagittal projection of the CBCT. Said length is measured in millimetres (Fig. 1).

**Figure 2:**
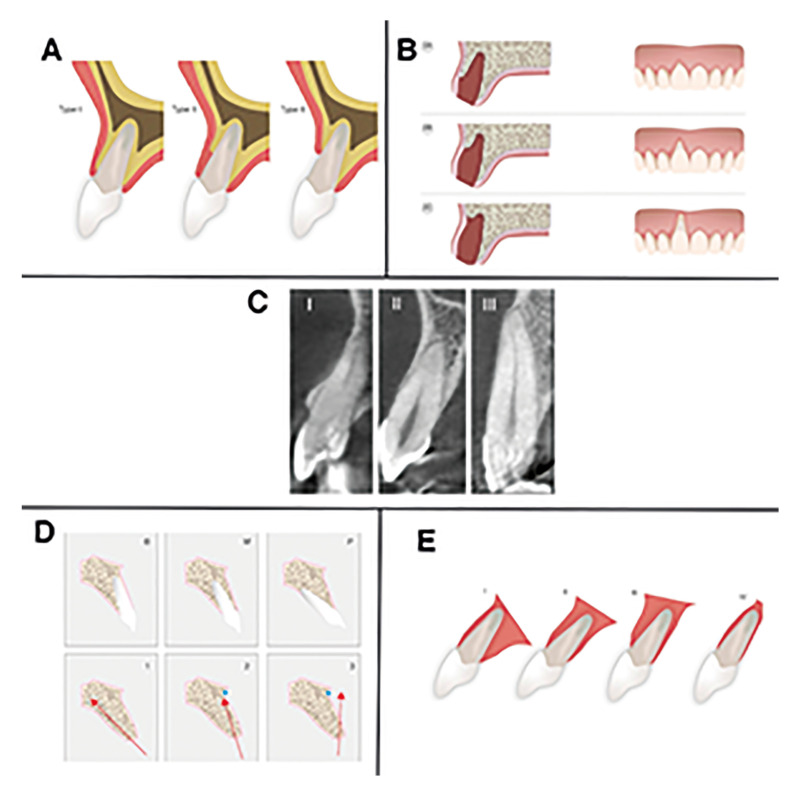
Different classifications of the type of sockets. (A) Types of sockets according to Elian *et al*.Type I: the buccal cortical is at a normal level in relation to the CEJ (cemento-enamel junction).Type II: partial loss of the buccal cortical. Type III: severe reduction of the cortical. (B) Types of sockets according to Chu *et al*.Type 2A: absence of the coronal third of the buccal cortical of the socket. Type 2B: absence of two thirds (middle to coronal third) of the buccal bone cortical.Type 2C: absence of the apical third of the buccal bone cortical. (C) Types of sockets according to El Chaar *et al*. Grade I: the buccal cortical is intact. It presents an adequate apical topography. Grade II: there is a dehiscence or fenestration in the buccal cortical that covers 25-50%. There is enough interproximal bone and it presents an adequate apical topography. Grade III: socket with inadequate apical topography, there is not enough interproximal bone or there is more than 50% loss of the buccal cortical. (D) Types of sockets according to Lau *et al*. Classification of the root positions: Type B: closer to the buccal alveolar surface. Type M: midway between the buccal and palatal alveolar surfaces. Type P: closer to the palatal alveolar surface. Classification of the angulations: Type 1: the root apex is angled towards the palatal side or parallel to the alveolus. Type 2: the apex of the root is angled towards the buccal side, with the long axis passing posterior to point A.Type 3: The root apex is angled towards the buccal side with the long axis passing anterior to point A. (E) Types of sockets according to Kan *et al*. Class I: the root is positioned against the buccal table. Class II: the root is centred in the middle of the alveolus without compromising the corticals both buccal and palatal at the apical third of the root. Class III: the root is positioned against the palatal corticals. Class IV: at least two thirds of the root are compromised with the buccal and palatal corticals.

Elian *et al*.´s classification (2007) (17): It divided the sockets into 3 types:

Type I: the buccal cortical and soft tissues were at a normal level in relation to CEJ (cemento-enamel junction). Type II: partial loss of the buccal cortical without loss of soft tissues. Type III: severe reduction of the buccal cortical and soft tissues (Fig. 2).

Chu *et al*.´s classification (2015) (18): Subdivided Elian *et al*.´s type 2 socket into 3 subtypes:

Type 2A: absence of the coronal third of the buccal cortical of the socket. Type 2B: absence of two thirds (middle to coronal third) of the buccal bone cortical. Type 2C: absence of the apical third of the buccal bone cortical (Fig. 2).

El Chaar *et al*.´s classification (2016) (12): The basis of this classification was the hard tissue (bone topography):

Grade I: the buccal cortical was intact (it is defined as intact if it did not present fenestrations or dehiscences, or whether it presented a loss of less than 25% of the height). The socket presented an adequate apical topography (defined as sufficient bone present apically at the time of extraction, at least 3-4mm for correct position of the implant). Likewise, there was an adequate interproximal bone (defined as not having interproximal bone loss or it is not greater than 2mm). Grade II: there was a dehiscence or fenestration in the buccal cortical which covers 25-50%. There was enough interproximal bone, and it presented an adequate apical topography. Grade III: deficient socket, which included any socket with inadequate apical topography, there was not enough interproximal bone or there was more than 50% loss of the buccal cortical (Fig. 2).

Lau *et al*.´s classification (2011) (9): This classification took into consideration the different positions and angulations of the root which provide a reference. This reference helped us avoid compromising the thickness of the buccal bone and its fenestration or perforation during implant placement:

Classification of positions:

Type B: closer to the buccal cortical surface. Type M: midway between the buccal and palatal corticals. Type P: closer to the palatal cortical

Angulations classification:

Type 1: the root apex was angled towards the palatal side or parallel to the socket. Type 2: the apex of the root was angled towards the buccal side, with the long axis being posterior to point A. Type 3: The apex of the root was angled towards the buccal side with the long axis being anterior to point A (Fig. 2).

Kan *et al*.´s classification (2011) (11): This classification was mainly based on the sagittal position of the root and the shape and size of the defect:

Class I: the root was positioned against the buccal cortical. There was a considerable amount of bone in the palatal area, which allows us to have primary stability. Class II: the root was centered in the middle of the socket without compromising the corticals both buccal and palatal in the apical third of the root. The amount of bone may not be adequate to achieve primary stability. Class III: the root was positioned against the palatal cortical. Therefore, the stability of the implant depends on it fitting in the available buccal bone. Class IV: at least two thirds of the root were compromised within the buccal and palatal corticals. (Fig. 2).

- Investigator´s calibration

Measurements of all CBCTs were performed by two of the investigators (J. P-M.; S.E-M) to observe the reliability of the ones performed by the main investigator. For categorical variables the kappa coefficient was used, giving a result of 0.82. In the case of continuous numerical variables, the intraclass correlation coefficient was used, which brought a result of 0.80. It was concluded that there was a high degree of agreement among investigators, therefore, there is a high reliability.

- Statistical analysis

The data of the variables were introduced in the Excel program of Microsoft Office 2019 Package (Microsoft Corporation, Washington, USA, 2013) and was analyzed with the SPSS 27.0 program for Windows (SPSS, Illinois, USA, 2019).

For data management, logic, range and consistency tests of results were used. For categorical variables, a descriptive analysis was carried out based on absolute, relative (frequency and percentage) and bivariate frequency measurement Tables with a chi-square test when the application conditions are not met. For the scale variables, the normality was verified with the Shapiro-Wilk test (*p*>0.1) and the visual analysis of the normal P-P graphs and box plots. If the application conditions were met, a descriptive analysis is made based on mean, standard deviation and bivariate with ANOVA parametric tests for independent samples. If the application conditions were not met, a descriptive analysis was made based on the median, interquartile range, and bivariate with non-parametric tests (Kruskal-Wallis test).

## Results

The sample consisted in 150 CBCTs, which corresponded to 87 women and 63 men. The total number of teeth examined were 485. The mean age of all subjects is 58.12±13.50 years (range 21-86 years old). The mean age of women was 58.03±13.95 years old and that of men was 58.73±12.97 years old.

Regarding the age ranges, we divided the patients into 4 groups: <45 years old, 45-54 years old, 55-64 years old and ≥65 years old. 15.1% of the teeth analyzed correspond to patients <45 years old, 21% to patients between 45 and 54 years old, 28% to patients between 55 and 64 years old and 35.9% to patients ≥65 years old.

Teeth examined were the upper teeth from right canine to left canine. In the group of women, 36.6% were central incisors, 32.6% lateral incisors and 30.8% canines. In the group of men, 37.8% were central incisors, 33.5% were lateral incisors and 28.7% were canines.

- Remaining bone support:

In the analysis of the remaining bone level, the data presented a lower percentage of remaining bone attachment in canines and central incisors, compared to lateral incisors, which presented 4.93% more than the first and 4.11% more than the second. These differences were not statistically significant. On the other hand, on the palatal surface there were significant changes from the statistical point of view. Lateral incisors presented less insertions levels than central incisors (5.92% less) and canines (2.82% less) (*p*<0.001). (Table 2).

When it comes to sex distribution, a lower percentage was observed in women, especially in the buccal areas of lateral incisors that presented a difference of 8.63% with men. In the palatal area, the greatest difference occurred in canines, with 6.06% (*p*=0.006), followed by lateral incisors with 4.46%. These differences were statistically significant for lateral incisors, both buccal and palatal (*p*= 0.002 and 0.024 respectively) (Table 2).

Regarding the differences by age, we noticed a gradual reduction of all the values with age, noting a slight increase between the groups of 55-64 years old and that of ≥65 years old. This effect occured in all the values, apart from the buccal cortical of central incisors, in which there was a reduction of 2.33%. The increase was 1.68% on the palatine face of central incisors, 2.62% on buccal areas of canines, 3.16% and 3.36% on buccal and palatal areas of lateral incisors respectively, being higher in canines in their buccal portion with 11.33%. These differences were only significant for the buccal areas of central incisors and canines (*p*=0.008 and 0.011 respectively) (Table 3).

A Pearson´s correlation test was performed to assess the relationship between the remaining support and the thickness of the buccal cortical at the three points. The test determined that the remaining support is a good predictor of the thickness of the buccal cortical at the most coronal point of central incisors (r=0.613) and canines (r=0.589).

- Assessment of the heights of the corticals and their thicknesses:

The assessment of the lengths and thicknesses of the corticals of every teeth yielded statistically significant results in all cases, except for postapical thicknesses. The buccal cortical is on average 1.98mm higher in canines and 1.25mm in central incisors, compared to lateral incisors. However, the thicknesses of the buccal cortical were lower in the case of canines, especially at the midpoint and apical point (0.61±0,55mm and 0.94±1,22mm respectively), in which the difference with the tooth that presented the greatest thickness in each case (central incisor for the midpoint and lateral incisor for the apical point) was 0.26mm and 0.4mm respectively. In the palatal cortical, central incisors were the ones with the greatest thickness at the most coronal point (1.66±0.86mm), distancing 0.22mm from the thinnest, which were the lateral incisors. At the other two points, canines had the thickest cortical (4.37±1.56mm and 8.86±2.78mm), distancing 1.01mm from lateral incisors at the midpoint and 2.54mm at the most apical point of the same tooth. Regarding the length from the apical point to the to the nostrils, lateral incisors presented the greatest distance (8.95±2.66mm), differing 0.07mm from central incisors and 1.41mm from canines. Canines had the greatest postapical thickness (12.50±3.83mm), being 0.17mm greater than central incisors and 0.79mm greater than lateral incisors (Table 3).

Table 3 shows the distribution of the sample by sex, in which there was a general decrease in heights and thicknesses of both buccal and palatal corticals in women, except in postapical lengths of central and lateral incisors (differences of 0.31mm and 0.28mm respectively) and the thickness of the buccal cortical at the midpoint of lateral incisors (difference of 1.02mm) which was greater in women. The rest of the values were slightly lower in women, with a difference compared to men from 0.22mm in the palatal cortical thickness of central incisors at the most coronal point; to 2.07mm in the postapical line of canines. All these differences were statistically significant, except for the thickness of the buccal cortical at the midpoint of central and lateral incisors, the postapical lengths in all the teeth analyzed and the thickness of the buccal corticals at the apical point and the most coronal thickness of the palatal cortical in canines (Table 3).

**Table 2:**
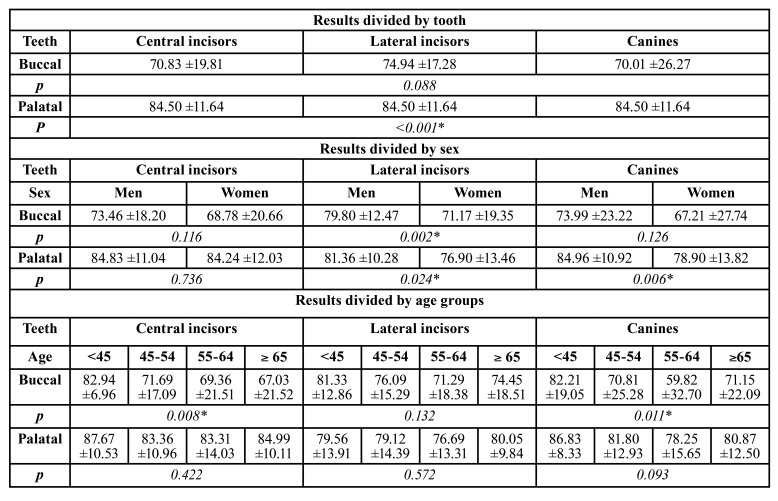
Arithmetic means and standard deviations of the percentages of buccal and palatal bone attachment levels of all the teeth examined, divided by type of tooth, sex and age group.

**Table 3:**
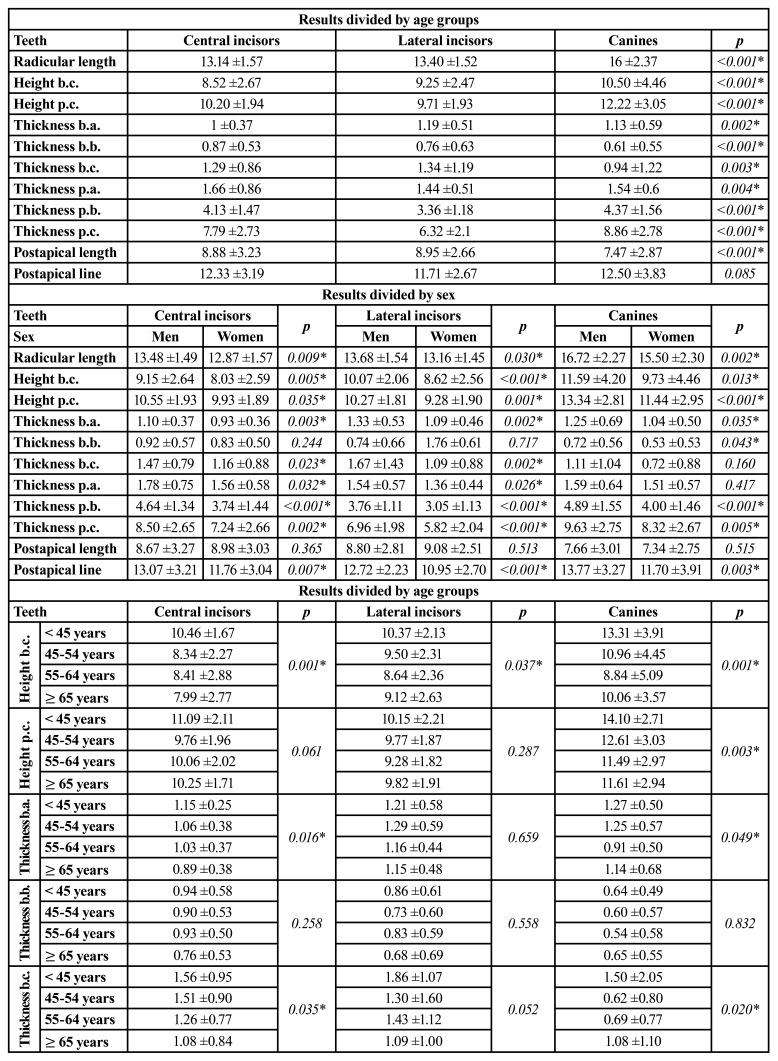
Arithmetic means and standard deviations of the lengths and thicknesses of the buccal and palatal corticals of the examined teeth and the lengths of their roots, measured in millimetres, divided by type of tooth, sex and age groups of patients.

**Table 3 cont.**
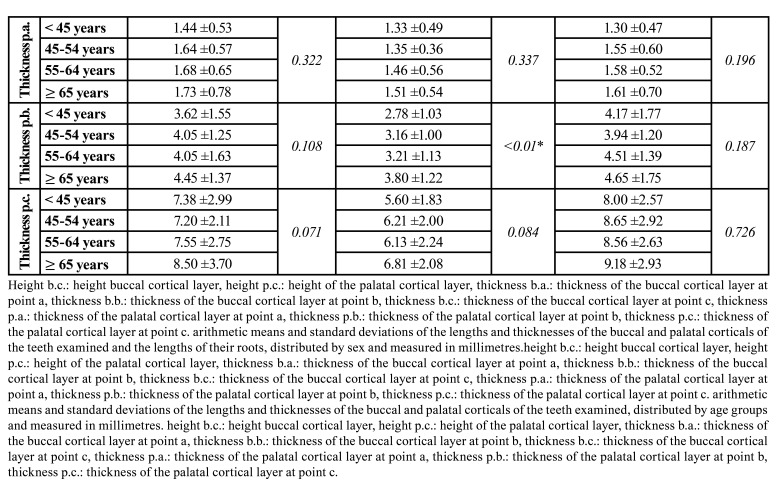
Arithmetic means and standard deviations of the lengths and thicknesses of the buccal and palatal corticals of the examined teeth and the lengths of their roots, measured in millimetres, divided by type of tooth, sex and age groups of patients.

Regarding the differences by age, a reduction in most of the values was observed between the group of <45 years old and 45-54 years old, and increased in one or two of the next groups. In the same way, an increase was observed in most of the values in the sample of patients ≥65 years old, compared to the group of 55-64 years old. Only a gradual reduction of the values divided by age was observed in the thicknesses of the buccal cortical of central and lateral incisors at the three points, as well as the postapical length in all analyzed teeth, the height of the buccal cortical in central incisors and the postapical line of canines. Nevertheless, the measurements that presented statistical significance were the height of the buccal cortical of all the teeth analyzed (*p*= 0.001 in central incisors and canines, and *p*=0.037 in lateral incisors), the height of the palatal cortical in canines (*p*=0.03), the thickness of the buccal cortical at the most coronal point of canines (*p*=0.049), the thickness of the buccal cortical at the most apical point of central incisors and canines (*p*= 0.035 and 0.020 respectively), and the mean thickness of the palatal cortical of the lateral incisors (*p*=<0.01) (Table 3).

- Distribution according to the different classifications of the types of socket:

The comparison between teeth was statistically significant except for Chu *et al*.´s classification (18). In Elian *et al*.´s classification (17), types I and III were higher in canines (13.1% and 10.3% respectively), than in the other teeth. Type II was the most prevalent in the 3 teeth (90.6%, 88.8% and 76.6%), being higher in central incisors. In Chu *et al*.´s classification (18), the most prevalent type in the 3 teeth was A (79.8%, 84.5% and 85.6%). Type B was found mainly in central incisors (19%) and type C in lateral incisors (1.4%). According to El Chaar *et al*.´s classification (12), types I and II were the most prevalent in the three groups (60% lateral incisors-49,4% central incisors for type I and 42.8% central incisors-30.3% canines), while type III occurs in a low percentage in the three teeth (11.7% cenines-4.4% lateral incisors). Regarding Lau *et al*.´s classification (9), the most prevalent type in the three groups of teeth was B2 (79.4% central incisors-77.9% canines), while B3 appears mostly in canines (17.9%). M1 and P1 types appeared mainly in lateral incisors (7.6% and 1.3% respectively) and B2 and M2 types appeared mostly in central incisors (79.4% and 1.1%). Kan *et al*.´s classification (9) showed the highest prevalence of socket type I in all the teeth analyzed (93.9%, 87.5% and 97.9%), the other three types appeared mostly in lateral incisors (8.1%, 1.3% and 3.1%).

The comparison by gender was only statistically significant for Chu *et al*.´s classification (18) (*p*=0.002) and that of El Chaar *et al*. (12) (*p*=0.001); both in lateral incisors. In the first one, we saw that type A was more prevalent in men (96.8%), while types B and C were more prevalent in women (22.5% and 2.5% respectively). Likewise, in El Chaar *et al*.´s classification (12), type I was more prevalent in men (75.7%) and types II and III are more prevalent in women (45.6% and 6.7% respectively).

The age distribution yielded more results which were statistically significant. Elian *et al*. (17) classification was significant for central incisors (*p*=0.031), for lateral incisors (*p*=0.009) and for canines (*p*=0.003). We noticed that sockets type I present a higher prevalence in patients <45 years old (37.5% canines-16.7% central incisors), while type III were more prevalent in individuals in the groups of 55-64 years old and ≥65 years old (18.9%-5.6% and 8%-3.3%, respectively). El Chaar *et al*.´s classification (12) was significant for central incisors (*p*=<0.001) and for canines (*p*=0.012). In both cases we can see that type I was the most prevalent. In the group of patients <45 years old, it was present in a high percentage of observations (95.8% central incisors-76% lateral incisors), while types II and III were mainly present in the groups of subjects of 55-64 years old and ≥65 years old (Table 4).

**Table 4:**
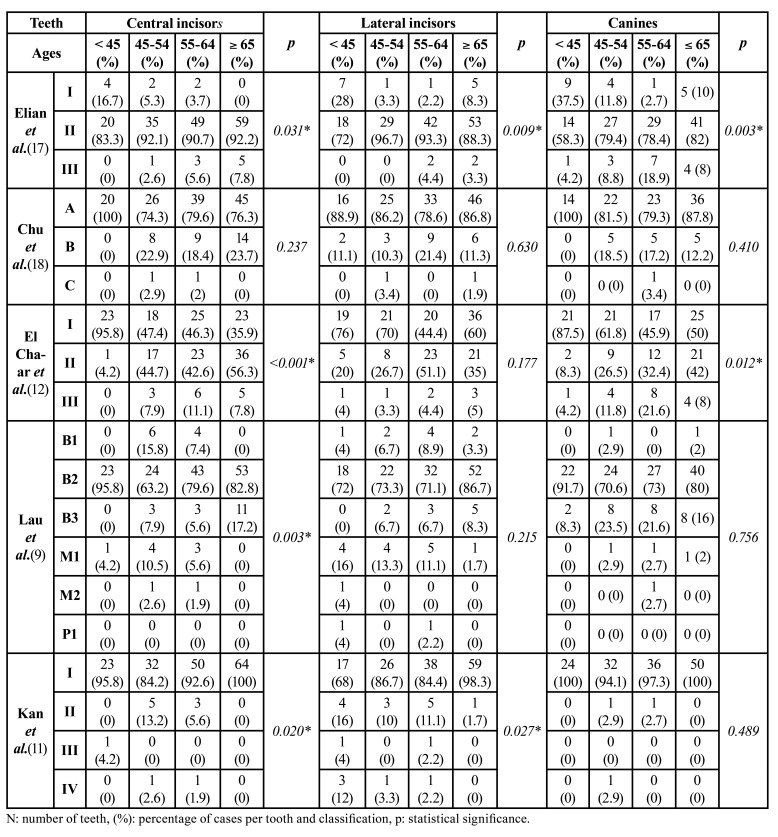
Distribution of the sample according to the different classifications, considering the age of the subjects.

Lau *et al*.´s (9) classification showed statistically significant results for the central incisors (*p*=0.003) in the groups distributed by age. Socket B2 presented the highest prevalence for all groups, while socket B1 was more prevalent in patients of 45-54 years old (8.9% central incisors and 0% canines). Socket B3 was mainly present in patients ≥ 65 years old (17.2% central incisors and 8.3% canines) and in the 4 age groups in canines. M1 type was more prevalent in patients between 45-54 years old in central incisors (10.5%) (Table 4).

Kan *et al*.´s classification (11), yielded statistically significant results for central (*p*=0.020) and lateral incisors (*p*=0.027), regarding the age division. In the central incisors, there was a high prevalence of type I in all groups, however, type II was present in 13.2% of individuals between 45-54 years old. In the lateral incisors we noticed that type I had the highest prevalence in individuals ≥65 years old (98.3%), while in the group of patients <45 years old, it was present in the 68%. Type II was present in the 16% of individuals who were <45 years, while in the group of ≥65 years old, it was present in 1.7% of the observations (Table 4).

## Discussion

When planning an immediate implant, we should bear in mind that the root position, and the remaining bone in the socket will influence the location of the osteotomy and the position of the implant in the 3 dimensions (19).

In Lau *et al*.´s study (9), type B2 socket is the most prevalent with 38.2% of all cases, which contrasts with our study, in which, although it is also the most prevalent one, the percentage of teeth with a B2 socket is 78.6%. Those authors gives a level II of difficulty to that type, because they present a thin buccal cortical both in the coronal and apical areas. Therefore, any pressure exerted to that area implies a risk of resorption, leading to the dehiscence of soft tissues in the long term. This would explain the physiological loss of hard and soft tissues on the buccal side in some cases of immediate implants. That is why, the author recommends not exerting pressure on the buccal cortical in these cases, even though it is difficult to achieve primary stability. In this sense, a study conducted by Menchini-Fabris *et al*. (20), showed a reduction of 22% of bone volume when the dental extraction is performed with a conservative method, compared to the conventional procedure. Consequently, these cases are sensitive to the technique. El Chaar *et al*. (12) advocate using guided bone regeneration techniques simultaneously with implant placement in this kind of socket.

B3 socket is the second most prevalent type, both in Lau *et al*.´s study (9), with 34.7%; which resembles to our study in which it appears in the 11% of all observations. Said authors classifies this type of socket with a level III, since it is the one that represents the greatest challenge to maintain a long-term aesthetics, as it has a thin buccal cortical. However, at the same time, the apex of the tooth is located very close to the buccal cortical, being in a position very anterior with respect to the contour of natural upper jaw. Consequently, the position of the implant in these cases will be compromised. Traditional strategy dictates that such teeth should be extracted, with or without concurrent bone regeneration. Moreover, the implant needs to be inserted several months later, because even if we do not achieve immediate implant placement, long-term soft tissue stability will be more predicTable. Furthermore, if we find enough bone in the palatal and apical areas, we can try to place the implant in a palatinized and buried position inside the bone to avoid touching the buccal cortical.

M1 type of socket are the third most prevalent type in our study (5.15%). In Lau *et al*.´s study (9) is present in 7.7% of the analyzed teeth. Those authors assigns a difficulty level I to it, since the roots are in the middle of the alveolar bone, and their angulation allows us to move away from the buccal cortical. This kind of socket will make it possible to achieve good primary stability and sufficient bone-to-implant contact for a good osseointegration. In the same way, the angulation of the implant will be adequate for the superstructure. All these conditions will produce a lower proportion of recessions and a better anatomy of the interdental papilla.

Kan *et al*.(19), in their study, show that the highest percentage of all the socket studied corresponds to type I, being 86.5% in central incisors, 76.5% in lateral incisors and 81% in canines. In our study, this type of socket is also the most prevalent, corresponding to percentages of 83.9%, 87.5%, and 97.9% in central incisors, lateral incisor, and canines, respectively. The second most prevalent socket according that the study are type IV, with percentages of 8%, 14% and 13% respectively. In our study, these sockets are the third most prevalent with percentages of 1.1%, 3.1%, and 0.7%, respectively. Type II socket, according to that author, presents percentages of 5%, 8.5% and 6% respectively. Our study shows the following percentages for this type of socket: 4.4%, 8.1% and 1.37% respectively, placing them in the second place.

Regarding the width of the buccal cortical in the superior aesthetic zone, it has been proved that having a cortical ≤ 1mm is a critical factor in terms of the extent of bone resorption, with this having an average of 7.5mm or 62.3% vertically and 0.8mm or 10.5% horizontally. In > 1mm corticals, there was a vertical resorption of 1.1mm or 9.1% and 0mm or 0% horizontally. These measurements were taken 8 weeks after a flapless extraction (21). In the present study, the percentage of teeth with a thickness of ≤1mm in the buccal cortical is 40.4% at the most coronal point, 65.6% at the midpoint and 40.6% at the most apical point. Among all the studied teeth, those with the thinnest buccal cortical, and therefore with the highest risk of resorption, are the canines. The 38.6% of all canines studied present a buccal cortical ≤1mm at the most coronal zone, 71.7% at the midpoint and 55.2% at the most apical point.

In a cross-sectional study based on CBCTs images, they concluded that the greater the distance between the CEJ and the bone crest is, the thinner the buccal cortical is (22). In this sense, in a review article conducted by Rojo-Sanchis *et al*. (23), concluded that the distance between the CEJ and the bone crest is greater in individuals older than 50 years. On the other hand, it has been proved that the buccal cortical is thinner in the most coronal and middle areas of the root, becoming thicker in the space between them. They also saw that women and the population over 50 years of age present thinner buccal corticals around the incisors and canines. In our study, as we indicated, the distance between the CEJ and the bone crest is greater as age increases in the three groups of teeth examined, only being statistically significant in incisors and canines on buccal areas. With regard to sex, this study has also shown that the buccal corticals are thinner in women in central incisors and canines, as well as in the age division in which statistical significance was found for central incisors and canines on the buccal areas.

In this study, a slight increase in the values of periradicular bone insertion in those patients ≥65 years old was proved. It is known that the effect of periodontal disease is cumulative, and the prevalence of periodontitis increases continuously between the ages of 30 and 80, with a sharp increase between the ages of 20 and 40, followed by stagnation. It has also been proved that patients who have access to dental services have a large percentage of surfaces which are not affected by periodontal disease, so periodontal destruction per se does not increase with age. Teeth lost as age advances are mainly multi-rooted ones, which are the ones with the highest risk of periodontal disease (24). In a study by Papanou *et al*. (25), they saw that the distance between the CEJ and the alveolar crest, in the group with the best periodontal status, varied between 1 and 1.5mm, but this value did not increase with age. The authors concluded that periodontal alterations caused by age did not have to manifest irretrievably with a decrease in periodontal insertion, nor loss of bone around teeth. Other study conducted by Bothelo *et al*. (26), concluded that patients with bruxism, therefore with occlusal erosions, had lower pocket depths and clinical attachment loss than patients without them. Their sample consisted in patients whose age was over 50 years old in 74.3% of cases. Thus, they found lower odds towards periodontitis and better periodontal clinical characteristics in these patients.

Finally, taking into account the results of this study, we can conclude that we will find worse levels of buccal bone attachment over upper central incisors and canines. Moreover, even though the length of the cortical bone over canines is greater, they are thinner, and therefore they present a greater risk of resorption; and together with the shorter postapical length, will make us plan the insertion of immediate implants more carefully. Although, there are statistically significant differences in the different measurements between men and women, only two classifications showed an increase in the complexity of the placement of immediate implants in lateral incisors in women, compared to men.

More observational and cohort studies are needed in this regard, taking soft tissues into account and the role played by the gingival phenotype in this process.

- Limitations of our study

One of our limitations is not having considered the gingival phenotype in the teeth analyzed, as well as the periodontal status and possible loss of soft tissue attachment.
